# Sleep disordered breathing and subjective excessive daytime sleepiness in relation to the risk of motor vehicle crash: the Toon Health Study

**DOI:** 10.1038/s41598-020-74132-7

**Published:** 2020-10-12

**Authors:** Ryotaro Matsuo, Takeshi Tanigawa, Kiyohide Tomooka, Ai Ikeda, Hiroo Wada, Koutatsu Maruyama, Isao Saito

**Affiliations:** 1 Department of Public Health, Faculty of Medicine, Juntendo University, Tokyo, 113-8421 Japan; 2 Department of Public Health, Juntendo University Graduate School of Medicine, Tokyo, 113-8421 Japan; 3 Laboratory of Community Health and Nutrition, Special Course of Food and Health Science, Department of Bioscience, Graduate School of Agriculture, Ehime University, Matsuyama, 790-0905 Japan; 4 Department of Public Health and Epidemiology, Faculty of Medicine, Oita University, Oita, 879-5593 Japan

**Keywords:** Public health, Epidemiology

## Abstract

Sleep disordered breathing (SDB) is a significant cause of motor vehicle crash (MVC). We conducted a prospective cohort study among 1047 Japanese community-dwellers to detect whether the presence or absence of subjective excessive daytime sleepiness (EDS) affect the association of SDB with a risk of MVC. SDB was assessed by a single airflow monitor which measured the respiratory disturbance index (RDI) during one-night, and participants were classified into the SDB group (RDI ≥ 10) and non-SDB group (RDI < 10). Subjective EDS was defined as Japanese version of Epworth Sleepiness Scale scores ≥ 11. A follow-up questionnaire five years after the baseline ascertained history of MVC over the period. Multivariable logistic regression analysis examined the association between SDB and MVC after stratification by subjective EDS. The multivariable-adjusted odds ratios (95% confidence interval) for MVC among the female SDB group were 1.66 (1.05–2.63) compared with the non-SDB group, and this association was more evident in females without subjective EDS [1.84(1.02–3.32)], but not among those with subjective EDS. There was no significant association in males. These findings indicate that SDB screening should be recommended regardless of subjective EDS to prevent SDB-related MVC among general population, particularly in females.

## Introduction

Sleep disordered breathing (SDB) is characterized by episodes of apneas and hypopneas during sleep and this condition is widely prevalent in the general population^1^. The prevalence of SDB (apnea–hypopnea index (AHI) ≥ 15) was 13% of men and 6% of women in the U.S.^2^, and 27.1% of men and 11.1% of women in Japan^3^. Clinical evidence suggests that SDB is a risk factor for motor vehicle crashes (MVCs)^4–6^, and a meta-analysis concluded that patients with SDB have a crash risk approximately 2.5 times that of individuals without SDB^5^. Moreover, the treatment with continuous positive airway pressure reduces the risk of MVCs^7^. Thus, early detection and prevention of SDB carries safety significance^8^.

Also, subjective excessive daytime sleepiness (EDS) is widely known as a risk factor for MVCs^9^, and is one of the diagnostic criteria of sleep apnea syndrome^10^. However, a previous study reported no significant difference in AHI score between the SDB patients with and without EDS^11^. Other study also reported that there are relatively many SDB patients who do not have subjective EDS in general population^12^; the reason for this discrepancy may be due to subjective assessment of EDS being underestimated by chronic sleep deprivation^13^ and differences among individuals^11^.

While a large number of studies have assessed the relationship between SDB and MVC, only a prospective cohort study examined the association of sleep apnea with or without subjective EDS on the risk of MVC^14^. The aim of this study was to examine whether individuals with SDB were at increased risk of MVCs, independent of subjective EDS in a prospective cohort study among Japanese community dwelling population.

## Results

The sex-specific characteristics of participants according to respiratory disturbance index (RDI) categories are shown in Table 1. Individuals with SDB, both males and females, were significantly older than those of the non-SDB group. The incidence of MVC tended to be higher among females with SDB, albeit insignificantly (*P* < 0.07).

**Table 1:** Sex-specific characteristics of the study participants according to the category of respiratory disturbance index (RDI).

	Males			Females		
	RDI, events/h		*P* value	RDI, events/h		*P* value
	< 10	≥ 10		< 10	≥ 10	
N	123	257	–	391	276	–
Age (years)	57.3	60.7	< 0.01	52.2	57.3	< 0.01
BMI (kg/m^2^)	23.3	23.9	0.02	22.1	23.4	< 0.01
JESS (score)	8.7	8.0	0.16	8.6	8.75	0.64
Sleeping duration (h)	6.6	6.8	0.18	6.5	6.4	0.69
Use of sleeping medications (%)	8.1	4.7	0.18	8.7	9.8	0.63
Current drinker (%)	76.4	77.4	0.83	48.1	42.0	0.12
Current smoker (%)	19.5	14.8	0.24	4.3	2.5	0.22
Diabetes mellitus (%)	10.6	13.2	0.46	5.9	7.6	0.38
MVC (%)	16.3	14.4	0.64	11.8	16.7	0.07

Mann–Whitney U test for continuous variables.

Chi-squared test for categorical variables.

*BMI* body mass index, *JESS* Japanese version of Epworth Sleepiness Scale, *MVC* motor vehicle crash.

Table 2 shows the results of multivariable-adjusted odds ratio (OR) (95% confidence interval (CI)) for MVC according to Japanese version of Epworth Sleepiness Scale (JESS) categories. The multivariable-adjusted ORs (95% CIs) of MVC among participants of the EDS group were 1.47 (0.80–2.72) for males, and 1.50 (0.94–2.40) for females, compared with non-EDS groups.

**Table 2:** Odds ratios (95% confidence intervals) of MVC according to JESS category.

	Males		Females	
	JESS, score		JESS, score	
	< 11	≥ 11	< 11	≥ 11
Participants, n	279	101	458	209
Cases, n	37	20	56	36
Model 1	1.00	1.62 (0.88–2.94)	1.00	1.49 (0.95–2.40)
Model 2	1.00	1.47 (0.80–2.72)	1.00	1.50(0.94–2.40)
Model 3	1.00	1.46(0.79–2.70)	1.00	1.43(0.91–2.27)

Model 1: adjusted for age.

Model 2: adjusted for age and sleep duration.

Model 3: adjusted for age and diabetes mellitus.

*JESS* Japanese version of Epworth Sleepiness Scale, *MVC* motor vehicle crash.

Table 3 shows the results of multivariable-adjusted OR (95% CI) for MVC according to RDI categories. The multivariable-adjusted ORs (95% CIs) of MVC among participants of the SDB group were 0.93 (0.51–1.70) for males, and 1.66 (1.05–2.63) for females, compared with non-SDB groups (*P* for interaction = 0.38). This association was also examined after stratification by the presence or absence of EDS (Table 4). The multivariable-adjusted ORs (95% CIs) of MVC among participants of the SDB group were 1.84 (1.02–3.32) for females free of EDS, whereas 1.44 (0.69–3.00) for females with EDS, compared with the respective participants of the non-SDB groups. The results also showed a borderline significant effect for EDS on the association between SDB and MVC (*P* for interaction = 0.07). No such association was found in males.

**Table 3:** Odds ratios (95% confidence intervals) of MVC according to RDI category.

	Males		Females	
	RDI, events/h		RDI, events/h	
	< 10	≥ 10	< 10	≥ 10
Participants, n	123	257	391	276
Cases, n	20	37	46	46
Model 1	1.00	0.92 (0.50–1.67)	1.00	1.66 (1.05–2.62)
Model 2	1.00	0.93 (0.51–1.70)	1.00	1.66 (1.05–2.63)
Model 3	1.00	0.92(0.50–1.68)	1.00	1.65(1.05–2.61)

Model 1: adjusted for age.

Model 2: adjusted for age, sleep duration.

Model 3: adjusted for age and diabetes mellitus.

*MVC* motor vehicle crash, *RDI* respiratory disturbance index.

**Table 4:** Odds ratio (95% confidence intervals) of MVC according to RDI stratified by presence of excessive daytime sleepiness.

	Males		Females	
	RDI, events/h		RDI, events/h	
	< 10	≥ 10	< 10	≥ 10
**JESS < 11**				
Participants, n	85	194	265	193
Cases, n	11	26	27	29
Model 1	1.00	1.11 (0.51–2.40)	1.00	1.82 (1.01–3.27)
Model 2	1.00	1.14 (0.52–2.47)	1.00	1.84 (1.02–3.32)
Model 3	1.00	1.11(0.51–2.40)	1.00	1.82(1.01–3.28)
**JESS ≥ 11**				
Participants, n	38	63	126	83
Cases, n	9	11	19	17
Model 1	1.00	0.68 (0.25–1.84)	1.00	1.45 (0.70–3.03)
Model 2	1.00	0.68 (0.25–1.84)	1.00	1.44 (0.69–3.00)
Model 3	1.00	0.66(0.24–1.79)	1.00	1.47(0.70–3.08)

Model 1: adjusted for age.

Model 2: adjusted for age and sleep duration.

Model 3: adjusted for age and diabetes mellitus.

*RDI* respiratory disturbance index, *JESS* Japanese version of Epworth Sleepiness Scale, *MVC* motor vehicle crash.

## Discussion

The major finding of the present study is to demonstrate the significant association between SDB and risk of MVC in female participants, and this association was more evident among those without subjective EDS. A previous prospective cohort study of 3201 community residents in the United States showed an increase in OR for MVC by 15% for every 10-unit increase in AHI in the overall population and by 17% for every 10-unit increase in AHI in participants who did not report subjective EDS^14^. In this study, the significant association was found only among female in the whole analysis, but not among males. However, we could not find any significant effect modification of sex on the association between SDB and MVC. The possible reason of this sex difference was that the number of male participants in this study was relatively small to detect the association between SDB and MVC.

In this study, 76% of male and 70% of female participants with SDB did not have subjective EDS. A previous large community-based study; the Sleep Heart Health Study also reported that 54.3% of their SDB participants did not have subjective EDS respectively even among those with moderate to severe SDB (AHI ≥ 15)^15^. These results suggest that there is a relatively higher proportion of individual with SDB who do not have EDS among general population.

Previous studies discussed the potential of subjective EDS as the cause of MVC^9^. In this study, subjective EDS was borderline significantly associated with increased risk of MVC in female participants. The causes of EDS are multifactorial, e.g., shortage of nocturnal sleep length, frequent sleep fragmentation^16^, dysfunction of circadian rhythm^17^, psychiatric disorders, diabetes^18^. Therefore, in this study, the independent association between SDB and MVC among females with EDS might be attenuated by these other factors which might causes EDS.

Furthermore, previous studies showed that the decline in vigilance was significantly associated with SDB^19^, and affected fatigue-related accidents, including MVCs^20^. On the other hand, other studies showed that discrepancy between awareness of subjective sleepiness and vigilance due to the consequences of chronic sleep deficiency has been reported^13^; suggesting that subjective EDS is less perceived to be due to cumulative sleep deficiency. In addition, a previous study reported that increased cortical activation during the task of auditory psychomotor vigilance significantly predicted poor driving performance among SDB patients^21^. Thus, a decline in vigilance, which affects driving performance, is significantly associated with SDB, rather than with subjective EDS. Our findings suggest that SDB is a risk factor for MVC, irrespective of subjective EDS. Therefore, screening for SDB even among individuals without subjective EDS might be significant for prevention of MVC.

The present study has several strength points. These include participants relatively high follow-up rate (69%) over 5 years, and it was conducted as a prospective cohort study among community residents and demonstrated the association of SDB with MVC after adjustment for potential confounding factors. Apart from these strengths, the study also has several limitations. First is that, the experience of MVC was based on retrospective self-administered data, therefore it potentially contains reporting bias. However, the Toon Health Study is administered in voluntary community residents. Compared with questionnaires administered by the employers as part of the medical examination (in Japan, employees undergo annual health checkups by the employers), there are no potential disadvantages or repercussions (e.g., losing jobs, increase in insurance premiums) by reporting MVC. Therefore, we believe that the participants declared their MVC experience correctly in the self-reported questionnaire in this study. Second is that one-channel flow-sensor device was used to assess the severity of SDB instead of polysomnography (PSG). However, the reliability and validity of the device was fairly good and quite comparable with full PSG tests^22,23^. Thirdly, although there might be other potential confounding factors on the association between EDS and MVC, e.g., dysfunction of circadian rhythm^17^, psychiatric disorders^18^, these variables were not available in this study. Further studies are needed in order to consider these issues.

In conclusion, we have demonstrated in the present study a significant association between SDB and MVC among females, and this association was more evident among those without EDS than those with EDS. SDB screening is strongly recommended regardless of EDS to prevent SDB-related MVC among community dwelling population.

## Methods

### Study subject

This study was conducted as a part of the Toon Health Study which is a community-based prospective cohort study to characterize the risk factors for cardiovascular disease in Toon City, Ehime prefecture, Japan^24,25^. In the present study, we recruited participants from approximately 22,000 residents of Toon city aged ≥ 30 to < 80 years using newspaper advertisements, posters, and direct invitations. At the baseline survey from 2009 to 2012, 2032 participants were enrolled in this study and 1395 participants were followed up approximately 5 years later. Of these, participants with incomplete data on overnight airflow monitor (n = 153) were excluded. We also excluded non-drivers (n = 166), those with a history of stroke (n = 27) and participants with missing data on sleep duration (n = 2). Thus, the data of 1047 (males = 380, females = 667) individuals were eligible for analysis.

The study protocol was approved by The Institutional Review Board of Ehime University Graduate School of Medicine and the Ethics Committee of Juntendo University, and written informed consent was obtained from each study participant. All methods in this study were performed in accordance with the relevant guidelines and regulations.

### Motor vehicle crash history

In a self-administered questionnaire completed approximately 5 years after the baseline, the participants were asked “Do you usually drive?” Those who responded affirmatively were also asked questions on MVC; “Have you ever had an accident while driving related to sleepiness in the past five years?” and “Have you ever had an accident while driving regardless of sleepiness in the past five years?” Participants who answered affirmatively for either of these two questions were defined as those who have had MVC during the five-year period.

### Assessment of sleep disordered breathing

The participants in this study completed a general sleep questionnaire and screening of SDB. To determine the presence or absence of SDB, a single-channel portable monitor; “Somnie” (NGK Spark Plug Co., Nagoya, Japan)^22,23^ was used during one night of sleep at home. The device records nasal/oral airflow with a polyvinylidene fluoride film sensor taped below the nose and has the capacity to store 24 h recording of the airflow signal. The morning after the recording, the participant completed a self-administered questionnaire about sleep state the night before. The data stored in the “Somnie” were subsequently analyzed by the “Flow” software (Institute of Sleep Health Promotion, Tokyo, Japan), which uses short-time power spectral analysis to compute the RDI. In this study, the criterion for SDB was defined by RDI level of 10 events per hour, and participants were classified as either SDB group or non-SDB. We have previously validated the “Somnie” device and confirmed that this RDI cutoff value represents AHI of ≥ 15 events per hour as determined by full PSG; with a corresponding sensitivity and specificity of 0.91 and 0.82^22^. We showed the prevalence of SDB among 5320 Japanese males by using the single-channel portable monitor^26^.

### Assessment of covariates

Height and weight were measured in stocking feet with light clothing. BMI was calculated as weight (kg) divided by the square of height (m^2^). Trained dietitian asked the participants to assess their drinking (never, former, or current drinker) and smoking (never, former or current smoker) habits. To assess EDS, each participant completed the JESS questionnaire, which is the sum of the scores of eight items (each from 0 to 3, total score range, 0–24). A JESS score of ≥ 11 represented the presence of EDS^27^. Sleep duration and use of sleeping medications were assessed using part of the Pittsburg Sleep Quality Index^28^. We also measured serum glucose levels during fasting and after the oral glucose tolerance test. All participants were required to fast for at least 10 h before the oral glucose tolerance test. The reference starch solution (Toleran-G75, Ajinomoto Pharma Co., Ltd., Tokyo, Japan.) containing 75 g of glucose was orally administered in a fasting state. Venous blood samples were obtained at baseline, 1 and 2 h after the ingestion of 75 g glucose. Diabetes mellitus was defined as either a fasting serum glucose level of ≥ 7.0 mmol/L, a 2 h post-load glucose level of ≥ 11.1 mmol/L or those receiving anti-diabetic medication.

### Analysis

In this study, we conducted sex-stratified analyses, because the proportion of RDI ≥ 10 events per hour was different between the two sexes (males = 69.2%, females = 41.6%). To analyze the differences between participants, the Mann–Whitney U test was used for continuous variables and chi-square test was used for dichotomous variables according to the RDI categories. Multivariable logistic regression analysis were used to calculate the ORs and 95% CIs for the MVC during the 5 years period according to JESS and RDI categories at baseline. Furthermore, stratified analysis was performed by the presence of EDS. The interaction of SDB with the presence of EDS in relation to MVC was tested using cross-product terms of these variables in the logistic regression model. Multivariable adjusted models were conducted using following confounding factors; age (year) in model 1, age (year) and sleep duration (h) in model 2, age (year) and diabetes mellitus (yes/no) in model 3. All statistical analyses were performed using SAS version 9.4 (SAS Institute, Inc., Cary, NC). All *P* values were two-tailed, and values < 0.05 were considered statistically significant.
